# Efficient Algorithms for Permutation Arrays from Permutation Polynomials

**DOI:** 10.3390/e27101031

**Published:** 2025-10-01

**Authors:** Sergey Bereg, Brian Malouf, Linda Morales, Ivan Hal Sudborough

**Affiliations:** Department of Computer Science, University of Texas at Dallas, P.O. Box 830688, Richardson, TX 75083, USA; brian.malouf@gmail.com (B.M.); lxm014000@utdallas.edu (L.M.); hal@utdallas.edu (I.H.S.)

**Keywords:** permutation Arrays, hamming distance, permutation polynomials

## Abstract

We develop algorithms for computing permutation polynomials (PPs) using normalization, so-called F-maps and G-maps, and the Hermite criterion. This allows for a more efficient computation of PPs for larger degrees and for larger finite fields. We use this to improve some lower bounds for M(n,D), the maximum number of permutations on *n* symbols with a pairwise Hamming distance of *D*.

## 1. Introduction

A *permutation array (PA)* is a set of permutations on *n* symbols. Let *n* be a positive integer and let σ and π be permutations over *n* symbols. σ and π have *a Hamming distance of at least D*, denoted by hd(σ,π)≥D, if σ(x)≠π(x) in at least *D* different positions *x*. A PA *A* has Hamming distance *D*, denoted by hd(A)≥D, if every pair of distinct permutations in *A* has Hamming distance at least *D*. The maximum number of permutations, over all PAs *A* on *n* symbols with hd(A)≥D, is denoted by M(n,D).

Let GF(q) denote the finite field over $q=p^{m}$ elements, where *p* is prime and m≥1. The prime *p* is called the *characteristic* of the field. A polynomial P(x) over GF(q) is a *permutation polynomial* (PP) if it permutes the elements of GF(q). Let $N_{d}\left(q\right)$ be the number of PPs of degree *d* over GF(q). Lidl and Mullen [1,2] posed the problem of computing $N_{d}\left(q\right)$. Dickson [3] characterized all PPs of degree up to 5 and degree 6 for polynomials with odd characteristics. Hou [4] gave a survey of recent results about PPs. Chu, Colbourn, and Dukes [5], using a table of all PPs of degree at most five given by Lidl and Mullen [1], counted the number of different PPs of degree at most 5. Shallue and Wanless [6] described those of degree 6. Li, Chandler, and Xiang [7] described PPs of degree 6 and 7 over a field of characteristic 2.

Much recent work has focused on computing large PAs with a given lower bound for their pairwise Hamming distance [5,8,9,10,11,12,13,14,15,16,17,18,19]. Chu et al. [5] showed that PPs can be used for lower bounds on M(n,D).

We find PPs of degree *d* over GF(q) to obtain PAs with a large Hamming distance. Let P(x) and Q(x) be two permutation polynomials of degree *d* over GF(q). Note that P(x) and Q(x) can agree in at most *d* points, because for every set of d+1 points there is one and only one polynomial of degree *d* that passes through them. So, the corresponding permutations must disagree in at least q−d positions. That is, the permutations have Hamming distance at least q−d. So, it follows that $M\left(q,q-d\right)\geq \sum_{k=1}^{d}N_{k}\left(q\right)$. Chu et al. [5] showed that, when *q* is a prime power, the following is true:

- If $q=2^{k}≢1\;\left(\mathrm{mod}\;3\right)$, then $M\left(q,q-3\right)\geq \left(q+2\right)q\left(q-1\right)\;\mathrm{and}\;M\left(q,q-4\right)\geq \frac{1}{3}q\left(q-1\right)$$\left(q^{2}+3q+8\right)$.
- If q≢1(mod3), then $M\left(q,q-2\right)\geq q^{2}$ ( In [5] a typo states q≢2(mod3).)

An early version of this paper appears in [20]. Because of its length, we have removed parts and refer the reader to [20] for missing details.

Recent work has also been performed on permutation rational functions (PRFs), which also give many large permutation arrays [11]. Let V(x) and U(x) be polynomials over GF(q), such that gcd(V(x),U(x))=1. Let $P^{1}\left(GF\left(q\right)\right)$ denote GF(q)∪{∞}. If the rational function V(x)/U(x) permutes the elements of $P^{1}\left(GF\left(q\right)\right)$, then it is called a permutation rational function. The lower bounds on the sizes of PAs described in [11] are summarized succinctly in Table 1. Full details and exact values can be found in [11]. For degrees 8 and 9, bounds are only known for q≤128. For *d* = 8, the bounds are of the form *c*·$q^{5}$ and *c*·$q^{6}$, for some c > 0, for M(q,q−d) and M(q+1,q−d), respectively. For *d* = 9, the bounds are of the form *c*·$q^{6}$ and *c*·$q^{7}$, for some c > 0, for M(q,q−d) and M(q+1,q−d), respectively.

Fan [21] obtained a classification of all permutation polynomials of degree 7 over GF(q) for any odd prime power q>7 up to linear transformations. Fan also [22] obtained a classification of all permutation polynomials of degree 8 over GF(q) for any odd prime power q>8 up to linear transformations and proved that there are no PPs of degree 8 for finite fields of order *q* when q>31. In addition, Fan [23] described all PPs of degree 8 over finite fields of characteristic 2.

Let

(1) $$P\left(x\right)=a_{d}x^{d}+a_{d-1}x^{d-1}+\ldots +a_{1}x+a_{0}$$

be a degree *d* permutation polynomial over GF(q). A brute force search for such a degree *d* permutation polynomials over GF(q) would require $O(dq^{d+2})$ time as there are d+1 coefficients, with *q* choices for each one, and, for each of these possibilities, one needs to examine the list of *q* values formed by the *d* terms of the polynomial on each element of GF(q) to see if the result is a permutation. Of course, the determination of whether P(x) over GF(q) is a PP may terminate as soon as P(a)=P(b), for distinct values a,b∈GF(q). This search technique is reminiscent of an odometer in that it tries all possibilities for coefficients, starting with low order terms first, and then proceeding to higher order terms. In this paper, we describe specific transformations of PPs, such as normalization and certain mappings, to restrict the search space, so that our odometer makes fewer turns than a brute force odometer. By using these methods we reduce the search time to $O(dq^{d-2}$). We have implemented the improved search algorithm, and provide some improved lower bounds for M(n,d) (Table 4), and Tables 3 and 5, which list values of $N_{d}\left(q\right)$ calculated by our program.

This paper is organized as follows. In Section 2, we discuss normalization of PPs. Normalization fixes three coefficients, which improves the search time to $O(dq^{d-1}$).

In Section 3, we review transformations that map PPs to PPs, which we call the F−map and the G−map. As the *F*-map allows us to restrict the range of another coefficient in a search for PPs, the time complexity is reduced to $O(dq^{d-2})$.

In Section 4, we describe uses of Hermite’s criterion, which allows the time complexity in some cases to be reduced again by an O(q) factor.

Finally, in Section 5, we present the results of our computations. We provide Table 3, in which we list specific values for $N_{12}\left(q\right)$, and Table 5, in which we list the total number of PPs for q≤97 and degree d,7≤d≤11. We also include Table 4, giving some new lower bounds for M(n,D).

*Notation*. In this paper, we use *p* to denote a prime and $q=p^{m}$ to denote a power of the prime *p* for some m≥1. We use *d* (2≤d<q) to denote the degree of a PP $P\left(x\right)=a_{d}x^{d}+a_{d-1}x^{d-1}+\ldots +a_{1}x+a_{0}$ over the finite field $GF\left(p^{m}\right)=GF\left(q\right)$ with field characteristic *p*. Throughout this paper and in the tables at the end, we use the following notation for the elements of GF(q). GF(q) has q−1 non-zero elements, all of which can be listed as $t^{0},t^{1},\ldots ,t^{q-2}$, where t≠0 represents a generator of the multiplicative group of nonzero elements of GF(q). We use the notation $t^{0}=1,t^{1}=2,\ldots ,t^{q-2}=q-1$. The elements of a finite field $GF(p^{m})$ can also be described as degree *m* polynomials with coefficients from GF(p) [24]. PPs can easily be converted from one notation to the other.

## 2. Normalization

Normalization facilitates our search for degree *d* PPs over a finite field GF(q) by fixing certain coefficients. It is known that any PP can be transformed into a normalized PP (nPP) by certain algebraic operations, which we describe shortly.

We describe nPPs by considering three cases based on the degree *d* of the polynomial, the characteristic *p* of the finite field, and whether *p* divides *d*. Let P(x) be a degree *d* polynomial with the coefficients as shown in statement (1) earlier. In all three cases, we have $a_{d}$ is fixed at 1 and $a_{0}$ is fixed at 0. In addition, when *p* does not divide *d*, $a_{d-1}$ is fixed at 0 [1].

In this paper, we use the names *c-normalization* to denote the case where p∤d, *m-normalization* when p>2 and p∣d, and *b-normalization* when p=2 and p∣d. In all three cases of normalization, searching for nPPs takes $O(dq^{d-1})$ time as three coefficients (positions) are fixed in the odometer process. Table 2 summarizes the three types of normalization.

Here are some examples of normalized PPs:

- The degree-9 PP $x^{9}+2x^{7}+3x^{5}$ over $GF(5^{2})$ is c-normalized, as $a_{9}=1$, $a_{8}=0$ and $a_{0}=0$.
- The degree-6 PP is $x^{6}+x^{5}+x^{3}+5x^{2}+5x$ over $GF(3^{2})$ is m-normalized, as $a_{6}=1$, $a_{4}=0$ and $a_{0}=0$.
- The degree-10 PP $x^{10}+x^{9}+x^{7}+26x^{5}+30x^{4}+21x^{2}+31x$ over $GF(2^{5})$ is b-normalized, as $a_{10}=1$, $a_{6}=0$ and $a_{0}=0$ (with i=3 and $r=2^{i}-1=7$).

An nPP is a representative of an equivalence class. We can make a more efficient search algorithm by searching for nPPs and the equivalence class they represent, rather than searching directly for PPs. The normalizationoperations for a polynomial P(x) are as follows: (i) multiplying P(x) by a non-zero constant a, (ii) adding a constant c to P(x), and (iii) adding a constant b to the argument *x* of P(x). They can be summarized simply by the statement that, if P(x) is a PP over GF(q), then aP(x+b)+c is a PP over GF(q).

The following result was given in [20]. The proof is included here.

**Theorem 1.** 
[20] *Any PP P(x) where the degree d is a multiple of the field characteristic p>2, can be transformed to an m-normalized PP Q(x) by the normalization operations.*

**Proof.**  Let $P\left(x\right)=a_{d}x^{d}+a_{d-1}x^{d-1}+\ldots +a_{1}x+a_{0}$, and, for some a,b,c∈GF(q) with a≠0, let the following be true:

$$\begin{array}{cc} Q(x) & =aP(x+b)+c\\ \; & =aa_{d}{\left(x+b\right)}^{d}+aa_{d-1}{\left(x+b\right)}^{d-1}+aa_{d-2}{\left(x+b\right)}^{d-2}+\ldots +aa_{1}\left(x+b\right)+aa_{0}+c\\ \; & =b_{d}x^{d}+b_{d-1}x^{d-1}+b_{d-2}x^{d-2}+\ldots +b_{1}x+b_{0}, \end{array}$$

Observe that the degree *d* term of Q(x) has the coefficient $b_{d}=aa_{d}$. If we choose *a* to be the multiplicative inverse of $a_{d}$, then the degree *d* coefficient of Q(x) will be 1.

If $a_{d-1}=0$, then $b_{d-1}=0$, and the desired property is true. So, suppose that $a_{d-1}\neq 0$, and consider $b_{d-2}$ in Q(x). Since *d* is a multiple of *p*, the expansion of $a_{d}{\left(x+b\right)}^{d}$ will derive nonzero coefficients only for terms whose degrees are multiples of *p*. Since p>2, this means that (d−2)∤p, so $a_{d}{\left(x+b\right)}^{d}$ will have a coefficient of 0 for the degree d−2 term. Hence, $b_{d-2}$ is calculated solely by the expansion of $aa_{d-1}{\left(x+b\right)}^{d-1}+aa_{d-2}{\left(x+b\right)}^{d-2}$.

The expansion of $aa_{d-1}{\left(x+b\right)}^{d-1}$ will produce a term of degree d−2 with the coefficient $aa_{d-1}b^{\prime }$ where $b^{\prime }=\sum_{1}^{d-1}b$. The expansion of $aa_{d-2}{\left(x+b\right)}^{d-2}$ will produce a term of degree d−2 with the coefficient $b_{d-2}=aa_{d-2}$. Therefore, the coefficient of $x^{d-2}$ in Q(x) is $b_{d-2}=aa_{d-1}b^{\prime }+aa_{d-2}=a\left(a_{d-1}b^{\prime }+a_{d-2}\right)$. Additionally, by algebra, $b_{d-2}$ is zero if $a_{d-1}b^{\prime }+a_{d-2}=0$. Since $a_{d-1}\neq 0$ and d−1 is not a multiple of *p*, we can choose *b* such that $b^{\prime }$ is the additive inverse of $a_{d-2}/a_{d-1}$, making $b_{d-2}=0$ in Q(x). So, in $Q\left(x\right),\;b_{d}=1,b_{0}=0,$ and either $b_{d-1}=0$ or $b_{d-2}=0$.

If we choose *c* to be the additive inverse of the constant term of aP(x+b), then the constant term becomes zero. So, we achieve m-normalization. □

The following result was shown in [20].

**Theorem 2** 
([20]). *Any PP P(x) over $GF(2^{m})$ for some m≥2, and 2∣d can be transformed to an b-normalized PP Q(x) by the normalization operations, except when $d=2^{i}-2$, for some i≥2.*

When the degree *d* of a PP is not a multiple of the field characteristic *p*, the PP can be c-normalized. As shown below, for any such PP P(x), the there is a unique triple (a,b,c) such that aP(x+b)+c is c-normalized. Moreover, each equivalence class contains exactly one nPP, and each PP belongs to exactly one equivalence class. These properties allow us to count, for a given *q* and *d*, the number of PPs in each equivalence class.

**Observation 1**  *Let* P(x) *be a degree d PP with characteristic p, where* p∤d*. Then, the triple* (a,b,c) *such that* aP(x+b)+c *is c-normalized is unique.*

**Proof.**  Let Q(x)=aP(x+b)+c such that Q(x) is c-normalized. The coefficient of its degree-*d* term is $aa_{d}=1$. Hence $a=a_{d}^{-1}$. The degree-(d−1) term of Q(x) is $\left(aa_{d-1}+db\right)x^{d-1}=0$, so $b=-aa_{d-1}/d$. (Note that d≠0). Finally, since the constant term of Q(x), namely Q(0), is 0, *c* is uniquely determined by the constant term of aP(x+b). □

Let Q(x) be an nPP of degree *d*. The equivalence class under the relation R containing Q(x), denoted by [Q], is the following set:

[Q]={aQ(x+b)+c|a,b,c∈GF(q)anda≠0}.

Note that, if a triple (a,b,c) is such that aP(x+b)+c=Q(x) is normalized, then the triple $\left(\alpha ,\beta ,\gamma \right)=\left(a^{-1},-b,-\frac{c}{a}\right)$ transforms Q(x) into P(x).

**Lemma 1.** 
*Let Q(x) be a c-normalized nPP of degree d<q where p∤d. Then, all $q^{2}\left(q-1\right)$ polynomials in [Q] are different.*

**Proof.**  Let $Q\left(x\right)=a_{d}x^{d}+a_{d-1}x^{d-1}+\ldots +a_{1}x+a_{0}$, with $a_{d}=1,a_{d-1}=0$ and $a_{0}=0$. One needs to show that, if $\left(\alpha ,\beta ,\gamma \right)\neq \left(\alpha^{\prime },\beta^{\prime },\gamma^{\prime }\right)$, then $\alpha Q\left(x+\beta \right)+\gamma \neq \alpha^{\prime }Q\left(x+\beta^{\prime }\right)+\gamma^{\prime }$. We show the contrapositive. Assume that the polynomials P(x)=αQ(x+β)+γ and $P^{\prime }\left(x\right)=\alpha^{\prime }Q\left(x+\beta^{\prime }\right)+\gamma^{\prime }$ in [Q] are equal. As the degree-*d* terms of P(x) and $P^{\prime }\left(x\right)$ are equal, it must be that $\alpha =\alpha^{\prime }$. Since the degree-(d−1) terms are equal, $\alpha \left(a_{d-1}+a_{d}db\right)=\alpha^{\prime }\left(a_{d-1}+a_{d}db^{\prime }\right)$. Then, as $\alpha =\alpha^{\prime }$, $a_{d-1}+a_{d}d\beta =a_{d-1}+a_{d}d\beta^{\prime }$ and $d\beta =d\beta^{\prime }$ and $\beta =\beta^{\prime }$ (since *d* is not a multiple of *p*). Let *e* be the lowest degree term of αQ(x+β). Then, the lowest degree terms of P(x) and $P^{\prime }\left(x\right)$ are e+γ and $e+\gamma^{\prime }$, respectively. Thus, $\gamma =\gamma^{\prime }$ and the claim follows. □

Note that Lemma 1 implies that each equivalence class contains one and only one nPP. Thus, when *d* is not a multiple of *p*, each equivalence class contains exactly $q^{2}\left(q-1\right)$ members (including the representative nPP). Note that the equivalence classes by definition are disjoint. If the number of nPPs is *k*, there are $kq^{2}\left(q-1\right)$ PPs. Similar statements apply for m-normalization and b-normalization.

We note that there is also an extended normalization operation, namely, multiplication of the variable, i.e., P(sx), for some nonzero constant *s*.

Extended normalization will come into play in Section 3. It is customary in the literature to refer to the normalization operations applied to a PP P(x) by aP(x+b)+c, where *a*, *b*, and *c* are elements of the finite field, with *a* being nonzero. References to extended normalization applied to a PP P(x), denoted by aP(sx+b)+c, where a,b,c, and *s* are elements of the finite field, with both *a* and *s* nonzero, appear, for example, in [21,22,23], and have been called linear transformations. The equivalence relation defined by the extended normalization operations will be denoted by $R_{E}$.

## 3. Mapping nPPs to nPPs

We now describe the *F-map* and the *G-map*, two functions that map nPPs to nPPs. The *F*-map is the function that multiplies the degree (d−k) term of P(x) by $t^{k}$, for all 0≤k≤d, where t is a generator of the cyclic group of non-zero elements of GF(q). The *F*-map allows one additional coefficient to have its range severely restricted, as we shall see, resulting in an order of magnitude speedup in the search for PPs.

Let P(x) be a degree *d* polynomial, with coefficients as shown in statement (1) earlier. Then, the following is true:

$$F\left(P\left(x\right)\right)=t^{0}a_{d}x^{d}+t^{1}a_{d-1}x^{d-1}+\ldots +t^{k}a_{d-k}x^{d-k}+\ldots +t^{d-1}a_{1}x+t^{d}a_{0}=\sum_{k=0}^{d}t^{k}a_{d-k}x^{d-k}.$$

**Theorem 3** 
([20]). *If P(x) is a nPP over GF(q), then F(P(x)) is a nPP over GF(q).*

The iterates of the F-map, acting on, for example the coefficient $a_{d-i}$, yields the sequence of coefficients $t^{i}a_{d-i},t^{2i}a_{d-i},t^{3i}a_{d-i},\ldots ,t^{(q-1)i}a_{d-i}$. If gcd(i,q−1)=1, then these coefficients are all distinct and contain all of the non-zero elements of GF(q). That is, the following sequence:

$$F\left(P\left(x\right)\right),\;F^{2}\left(P\left(x\right)\right),\;\ldots \;,\;F^{q-1}\left(P\left(x\right)\right)$$

This is a cycle in which the coefficient of the $x^{d-i}$ term takes on each of the non-zero elements of GF(q). In this case, one need only consider the possibilities that the coefficient of the $x^{d}$ term is 0 or 1. Clearly, a coefficient 0 maps to itself under the F-map. Any other possible coefficient for the $x^{d}$ term is mapped to by the sequence of F-map iterates when $a_{d-i}=1$. This means that one can examine the two cases, (1) $a_{d-i}=0$, and (2) $a_{d-1}=1$. In case (2), if P(x) is a nPP, when $a_{d-i}=1$, then one can simply multiply the number of nPPs by q−1, and not explicitly consider nPPs with other non-zero choices for $a_{d-i}$. That is, the odometer for that coefficient can be fixed at 1.

On the other hand, if gcd(i,q−1)=m, where $\frac{q-1}{m}=s$, then there are *s* cycles, each of length *m*, such that the non-zero elements of GF(q) each appear once in one and only one of the *s* cycles. In this case, one only need consider the values 0,1,2,…,s for the coefficient $a_{d-i}$.

It should be noted that one can choose the coefficient $a_{d-i}$ for any i (2≤i≤d−1). In most cases, there is such an *i* such that gcd(i,q−1)=1.

As mentioned earlier, we have created a program that employs the efficiencies of normalization and F-maps and G-maps. We describe some of the improved results.

**Example 1.** 
*Consider degree-9 permutations of the form $P\left(x\right)\;=\;x^{9}+a_{7}x^{7}+a_{6}x^{6}+a_{5}x^{5}+a_{4}x^{4}+a_{3}x^{3}+a_{2}x^{2}+a_{1}x$ over $GF(5^{2})$. The form is restricted to possible c-normalized PPs, as $a_{9}=1$, $a_{8}=0$ and $a_{0}=0$. In this example, q−1=24. We observe that gcd(2,24)=2, gcd(3,24)=2, gcd(4,24)=4, gcd(5,24)=1, gcd(6,24)=4, gcd(7,24)=1, and gcd(8,24)=8. Our program would select i=5, as that is the smallest index i which gives one cycle under the F-map for all non-zero elements of GF(q). So, as d=9, $a_{d-5}=a_{4}$, and the program only considers P(x) with $a_{4}=0$ and $a_{4}=1$.*

*The program also calculates the number of nPPs in a given F-map cycle for any nPP Q(x) found. For example, for the family of permutations P(x) given above, with $a_{4}=0$, it finds that the nPP $Q\left(x\right)=x^{9}+2x^{7}+9x^{5}+22x^{3}+11x$ is an nPP over GF(24). Here, $a_{7}=2$, $a_{6}=0$, $a_{5}=9$, $a_{3}=22$, $a_{2}=0$, and $a_{1}=11$. As each of the non-zero coefficients in Q(x) is in a cycle of length 2 or a multiple of 2, the number of nPPs in the F-map cycle is $\frac{q-1}{2}=\frac{24}{2}=12$.*

Consider a finite field $GF(p^{k})$, where k>1, and a permutation P(x) over $GF(p^{k})$. The *G*-map is a function that raises each coefficient in P(x) to the power *p*.

Let P(x) be a degree d polynomial, with coefficients as shown in statement (1) earlier. Then, the following is true:

$$G\left(P\left(x\right)\right)=a_{d}^{p}x^{d}+a_{d-1}^{p}x^{d-1}+\ldots +a_{1}^{p}x+a_{0}^{p}=\sum_{k=0}^{d}a_{d-k}^{p}x^{d-k}.$$

The iterates of the G-map, acting on, for example, the coefficient $a_{d-i}$, yields the sequence of coefficients $a_{d-i},a_{d-i}^{p},a_{d-i}^{2p},\ldots ,a_{d-i}^{(m-1)p}$. This allows one to restrict the range of values considered by our odometer program for a coefficient $a_{d-i}$.

**Example 2.** 
*Consider degree-9 permutations over $GF(5^{2})$ of the form $P\left(x\right)\;=\;x^{9}+a_{7}x^{7}+a_{6}x^{6}+a_{5}x^{5}+a_{4}x^{4}+a_{3}x^{3}+a_{2}x^{2}+a_{1}x$. As stated earlier, the form is restricted to possible c-normalized PPs, as $a_{9}=1$, $a_{8}=0$ and $a_{0}=0$. In this example, q−1=24. The program also calculates the number of nPPs in a given G-map cycle for any nPP Q(x) found. For example, for the family of permutations P(x) given above, with $a_{4}=1$, it finds that the nPP $Q\left(x\right)=x^{9}+10x^{7}+8x^{5}+x^{4}+8x^{3}+17x$ is an nPP over $GF(5^{2})$. Observe that $G\left(Q\left(x\right)\right)=x^{9}+10^{2}x^{7}+8^{2}x^{5}+x^{4}+8^{2}x^{3}+17^{2}x$, which is not in the F-map cycle of Q(x), as the coefficient for $x^{4}$ remains 1, but other coefficients have changed. In fact, for each of the 24 nPPs in the F-map cycle of Q(x), applying the G-map to each gives a nPP that is not in the F-map cycle [20]. So, the program automatically calculates, in this example, that there are 48 nPPs corresponding to Q(x) by applying the F-map and G-map.*

Our examples have focused on c-normalization. We note that F-maps and G-maps can also be used for m-normalization and b-normalization.

**Theorem 4** 
([20]). *Let p be a prime and and let $q=p^{m}$ with m>1. If P(x) is a nPP over GF(q), then G(P(x)) is a nPP over GF(q).*

These two functions, the *F*-map and *G*-map, transform PPs into other PPs and nPPs into other nPPs. These functions can be applied sequentially. It is interesting to note that two different sequences of compositions can represent the same transformation. This is illustrated by the following diagram, which indicates that (G∘F)(P(x)) is the same as $\left(F^{p}\circ G\right)\left(P\left(x\right)\right)$, for all PPs P(x).


$$\begin{array}{ccc} P(x) & \overset{\;G\;}{\to } & G(P(x))\\ F↓ & & ↓F^{p}\\ F(P(x)) & \vec{\;\;G\;\;} & \left(F^{p}\circ G)(P(x)\right) \end{array}$$

The equivalence relations R and $R_{E}$, which we described in Section 2, allow a more efficient search for PPs by limiting the search to nPPs. More inclusive equivalence relations would optimize the search even further, by allowing the search to be restricted to representatives of equivalence classes. In this section, we introduce new equivalence relations, based on the *F*-map and the *G*-map and iterations of the maps, that merge equivalence classes, thereby compressing the search space considerably. We begin with an equivalence relation induced by the *F*-map.

**Definition 1.** 
*Let P(x) and Q(x) be PPs. If P(x) can be converted into Q(x) by some sequence consisting of normalization operations and F-map operations, then P(x) and Q(x) are related by $R_{F}$.*

We have seen that the iterates of the *F*-map form a cycle. Moreover, if P(x) is an nPP, then the *F*-cycle on P(x) is a cycle of nPPs.

**Lemma 2** 
([20]). *The equivalence relations $R_{F}$ and $R_{E}$ are the same.*

We can choose any nPP in an $R_{F}$ equivalence class to be the representative for the class. For convenience, we usually choose the term of largest degree which has the longest cycle under the F-map. Additionally, for each such cycle for that term, we choose the smallest coefficient in the cycle. for a specific chosen degree to be the representative. To illustrate, let P(x) be an nPP in some equivalence class, let the specific degree be d−k for some *k*, and consider the the coefficient of $x^{d-k}$, namely $a_{d-k}$. Let the multiplicative inverse of $a_{d-k}$ be $t^{ik}$ for some *i*. Suppose the length of the *F*-cycle on $a_{d-k}$ is q−1. Then, sequence $a_{d-k},\;t^{k}a_{d-k},\;t^{2k}a_{d-k},\;\ldots \;,t^{rk\;\mathrm{mod}\;(q-1)}a_{d-k}$ includes every nonzero element in GF(q). Specifically, for one element in the *F*-cycle, say the i-th one, $a_{d-k}t^{ik}=1$. In other words, in the nPP $F^{i}\left(P\left(x\right)\right)$, the coefficient of $x^{d-k}$ takes the value 1. So, in this case we choose $F^{i}\left(P\left(x\right)\right)$ to be the representative of the equivalence class, making the search for nPPs more efficient since the coefficient of $x^{d-k}$ can be fixed to 1. Notice that, if the length of the *F*-cycle on P(x) is less than q−1, then there may be a nPP with a nonzero ${\left(d-k\right)}^{th}$ coefficient, but not one with the ${\left(d-k\right)}^{th}$ coefficient equal to 1. In that case, if the cycle length is (q−1)/j, for some j>1, then one needs to search for nPPs with a ${\left(d-k\right)}^{th}$ coefficient equal to each of the values 1,2,3,…,j−1. Also, if there is an nPP whose ${\left(d-k\right)}^{th}$ coefficient is zero, it would be chosen as the representative for the equivalence class.

So, again, in our search for all permutation polynomials over GF(q) for a given *q*, we can reduce the search to the space of normalized PPs which are representatives of an *F*-map equivalence class. Specifically, when the degree of the polynomial is not a multiple of the field characteristic, we can restrict the search to polynomials $a_{d}x^{d}+a_{d-1}x^{d-1}+\ldots +a_{1}x+a_{0}$, where $a_{d}=1$, $a_{0}=a_{d-1}=0$, and $a_{i}$ ranges over all cycle representatives. We choose a value of *i*, so that the $i^{th}$ coefficient has the longest cycle. When the degree is a multiple of the field characteristic, and the characteristic is not 2, we restrict the search to polynomials $a_{d}x^{d}+a_{d-1}x^{d-1}+\ldots +a_{1}x+a_{0}$, where $a_{d}=1$, $a_{0}=0$, and one of the following cases (1) $a_{d-1}=0$, and (2) $a_{d-1}\neq 0$ and $a_{d-2}=0$. The statement is similar when the field characteristic is 2 and the degree of the polynomial is even.

For example, consider the nPP P(x) = $x^{8}+4x^{4}+16x^{2}+3x$ for $GF(2^{5})$, with the primitive polynomial $x^{5}+x^{3}+x^{2}+x+1$. As there are 31 non-zero elements in $GF(2^{5})$, and 31 is prime, the length of the *F*-cycle on P(x) is 31. The iterates of *G*-map on the coefficient 16 give the *G*-cycle {16,31,30,27,24}, which has a length of 5. So, [P(x)] denotes an equivalence class with 31∗5=155 nPPs. Although the *G*-map (unlike the *F*-map) does not allow, in general, an additional coefficient to be fixed, when used with the *F*-map, it does allow a more efficient search.

## 4. Using Hermite’s Criterion

The following is a statement derived from Hermite’s criterion [3,22,25].

**Theorem 5** 
([25]). *If f(x) is a PP over GF(q), then for each k, with 1≤k≤q−2, the sum of the coefficients of $x^{(q-1)i}$ in $f{\left(x\right)}^{k}$, over all positive integers $i\leq \lfloor \frac{deg(f{\left(x\right)}^{k})}{q-1}\rfloor$, must be 0.*

Theorem 5 can be used to fix another coefficient in a nPP beyond those already described.

For example, let $P\left(x\right)=x^{11}+a_{9}x^{9}+\ldots +a_{1}x+a_{0}$ be a nPP of degree 11 over $GF(2^{5})$. By Theorem 5, in $P{\left(x\right)}^{3}$ the coefficient of $x^{31}$ must be 0. As the coefficients $a_{11}$ and $a_{10}$ of P(x) are, respectively, 1 and 0, computing the coefficients of $P{\left(x\right)}^{3}$ by expansion, one finds that the coefficient of $x^{31}$ is simply $3a_{9}$. So, in searching for degree 11 nPPs over $GF(2^{5})$ one can fix the coefficient $a_{9}$ to be 0. That means, to summarize, in searching for degree 11 nPPs over $GF(2^{5})$, by normalization, one fixes coefficients $a_{11}$, $a_{10}$, and $a_{0}$ to 1, 0, and 0, respectively. By using Hermite’s criterion, one can fix $a_{9}$ to be 0 and, by the F-map, one can fix $a_{8}$ to be either 0 or 1. As far as we know, one needs to try all possible combinations of elements of $GF(2^{5})$ for the seven remaining coefficients. That is, $32^{7}=2^{35}$ different combinations, which is large, but smaller, of course, than $32^{8}$. Specifically, the computation of all these possibilities can be done in a few days, rather than in a few months.

In a similar manner, using Hermite’s criterion and the nPP P(x) = $x^{11}$ + $a_{9}x^{9}$ + … +$a_{1}x+a_{0}$, one can give additional examples:

(a) In $P{\left(x\right)}^{4}$ over GF(41), the coefficient of $x^{40}$ must equal 0, so $4a_{7}+6a_{9}^{2}=0$. That is, $a_{7}=\frac{-6a_{9}^{2}}{4}$, i.e., the value of $a_{7}$ depends on the value of $a_{9}$.
(b) In $P{\left(x\right)}^{5}$ over GF(53), the coefficient of $x^{52}$ must equal 0, so $5ba_{8}=0$, i.e., $a_{8}=0$.

Our search for all nPPs over a given finite field uses such examples from Hermite’s criterion for efficient computations. That is, the program allows a particular set of coefficients to be fixed to a set of chosen values. For example, in example (b) above, the program allows one to fix the coefficient $a_{8}$ to be zero. Clearly, fixing any set of coefficients to a specified set of values makes the odometer program terminate quicker.

The idea of fixing a set of chosen coefficients to a specified set of values also allows one to search for PPs in a distributed manner. For example, we computed all PPs of degree 13 over GF(19) using this approach. So, for polynomials of the form $P\left(x\right)=a_{13}x^{13}+a_{12}x^{12}+a_{11}x^{11}+\ldots +a_{1}x+a_{0}$, by c-normalization, we fixed three coefficients, namely $a_{13}=1$, $a_{12}=0$, and $a_{0}=0$. By the use of F-maps we fixed coefficient $a_{8}$ to two possible values, namely, 0 and 1. (We chose $a_{8}$, for the F-map, as $t^{13-8}=t^{5}$, and gcd(5,18)=1. That is, the F-cycle on $a_{8}$ has maximum length.) By choice, for the sake of using a distributed approach, we separately fixed $a_{1}$ to be each of the nineteen possible values in GF(19), and ran the program on each of these nineteen choices simultaneously, using different CPUs.

The result is $N_{13}\left(19\right)=884,354,582,646$. So, $M\left(19,6\right)\geq \sum_{i=1}^{13}N_{i}\left(19\right)=933,551,546,094$. This is a slight improvement over the previous lower bound of 933,426,695,689 [8].

## 5. Results

As stated in Section 1, a brute force search for degree *d* permutation polynomials over GF(q) requires $\Theta (dq^{d+2})$ time. Normalization operations are defined in [1]. For example, for PPs in which p∤d, the operation fixes three of the coefficients and, therefore, reduces the time to $\Theta (dq^{d-1})$. We improved the time bound to $O(dq^{d-2})$ by fixing an additional coefficient by the F-map and, in many cases, improved the time bound to $dq^{d-3}$ by the use of Hermite’s criterion. Our program also allows a specified set of coefficients to be fixed at specified values. This is needed in our use of the Hermite criterion and allows for the search to be distributed.

Our results are shown in Table 5, which lists the total number of PPs for q≤97 and degree *d*, where 7≤d≤11. For degrees d≤6, all PPs have been described; for example, in [5]. More recent work [6,7,21,22,23] gives all PPs of degree d≤8. We list some of these in Table 5 for completeness. Table 5 does not have columns for d≥12. However, we include some values for $N_{12}\left(q\right)$ in Table 3. This yields $M\left(17,5\right)\geq \sum_{i=1}^{12}N_{i}\left(17\right)=72,377,516,320$$,M\left(19,7\right)\geq \sum_{i=1}^{12}N_{i}\left(19\right)=49,196,963,448$, and $M\left(23,11\right)\geq \sum_{i=1}^{12}N_{i}\left(23\right)=14,341,972,920$.

We note that it was stated in [26] that 32 h were required to compute the RS code for q=32 and d=5; for q=32 and d=7, the authors were able to compute only the size of the set of PPs, not the set itself. Our program allows for the computation of all nPPs, for q=32 and d=7, in a few seconds and, for q=32 and d=8, in about 10 min.

Our results on the number of PPs also give some new lower bounds for M(n,D). These are given in Table 4 using $M\left(q,D\right)\geq \sum_{i=1}^{D}N_{i}\left(q\right)$, and the values of $N_{i}\left(q\right)$ given in Table 3, Table 5 and separately in the text. We note that the lower bound $M\left(n,d\right)\geq n!/q^{d-2}$, for a prime power q≥n and 2<d<n, is given in [8]. Here are some examples of computations to determine the competitiveness of our bounds:

- M(16,5): $\sum_{i=1}^{11}N_{i}\left(16\right)=5,112,053,760$. Previous lower bound in [8] is $\frac{16!}{16^{3}}=5,108,103,000$. Our bound is slightly better.
- M(17,6): $\sum_{i=1}^{11}N_{i}\left(17\right)=4,250,533,664$. A lower bound of 4,258,658,638 is given in [8], so our bound is not competitive.
- M(23,11): $\sum_{i=1}^{12}N_{i}\left(23\right)=14,341,972,920$. A lower bound of 14,353,040,302 is given in [9], so our bound is not competitive.
- M(25,15): $\sum_{i=1}^{10}N_{i}\left(25\right)=257,205,000$. A lower bound of 2,319,933,000 for M(25,16) is given in [11]. Since M(n,D)≥M(n,D+1) [5], our bound is not competitive.
- M(25,14): $\sum_{i=1}^{11}N_{i}\left(25\right)=6,766,439,400$. Using the previous item, we have M(25,14)≥M(25,16)≥2,319,933,000. So, our bound is better than the previous ones.
- M(81,72): $\sum_{i=1}^{9}N_{i}\left(81\right)=3,100,041,120$. A lower bound of M(81,73)=2,934,438,840 is given in [11], which also gives, of course, the same lower bound for M(81,72). Our bound is larger.

**Table 4:** Some new lower bounds for M(n,D).

*n*	*D*	M(n,D)≥	Previou
16	5	5,112,053,760	5,108,103,000 [8]
19	6	933,551,546,094	933,426,695,689 [8]
19	7	49,196,963,448	49,127,770,826 [9]
25	14	6,766,439,400	2,319,933,000 [11]
81	72	3,100,041,120	2,934,438,840 [11]

**Table 5:** Total number of PPs for q≤97 and degree *d*, where 7≤d≤11.

q/d	7	8	9	10	11
**11**	272,250	3,332,340	36,281,850		
**13**	233,220	2,798,640	33,948,720	442,144,560	
**16**	829,440	3,555,840	16,128,000	340,224,000	4,751,093,760
**17**	966,416	0	13,978,352	234,011,392	4,001,494,000
**19**	727,776	5,614,272	0	126,996,912	2,431,915,488
**23**	1,035,782	1,792,252	35,984,696	586,578,476	0
**25**	675,000	0	15,570,000	240,768,000	6,509,295,000
**27**	265,356	6,899,256	20,744,100	134,535,492	2,826,989,100
**29**	0	753,536	41,232,548	36,923,264	1,014,518,484
**31**	3,055,980	864,900	18,162,900	0	385,053,880
**32**	1,015,808	19,467,008	9,872,384	407,434,240	190,940,160
**37**	1,823,508	0	0	10,645,344	446,266,620
**41**	67,240	0	22,256,440	0	52,581,680
**43**	0	0	3,261,636	7,610,484	17,473,060
**47**	0	4,775,858	11,787,224	0	58,631,278
**49**	9,316,272	0	11,063,808	1,843,968	260,114,736
**53**	7,741,604	0	7,741,604	0	11,539,372
**59**	11,911,982	0	23,622,066	0	11,911,982
**61**	13,618,860	0	0	0	
**64**	0	326,462,976	0	85,155,840	43,610,112
**67**	19,850,358	0	0	0	
**71**	0	0	25,053,770	0	
**73**	28,009,224	0	0	0	
**79**	38,457,042	0	0	0	
**81**	42,515,280	0	3,057,853,680	0	
**83**	564,898	0	46,886,534	0	
**89**	62,037,272	0	62,037,272	0	
**97**	903,264	0	0	0	

Additional improved lower bounds can be obtained from those shown in Table 4 using inequalities, such as M(n−1,D)≥M(n,D)/n [5], or the operation of contraction [10].

## Figures and Tables

**Table 1:** Known asymptotic lower bounds for PAs obtained by PRFs for prime powers q≥10.

d	M(q, q − d)	M(q + 1, q − d)
4		$\Omega (q^{3})$
5	$\Omega (q^{4})$	$\Omega (q^{5})$
6	$\Omega (q^{4})$	$\Omega (q^{5})$
7	$\Omega (q^{5})$	$\Omega (q^{6})$

**Table 2:** Types of normalization for PPs, $P\left(x\right)=a_{d}x^{d}+a_{d-1}x^{d-1}+\ldots +a_{1}x+a_{0}$, of degree *d* with field characteristic *p*.

Normalization Type	Degree Restriction	nPP Properties
*c-normalization*	p∤d	monic, $a_{0}=0,a_{d-1}=0$
*m-normalization*	p∣d and p>2	monic, P(0)=0, either $a_{d-1}=0$ or $a_{d-2}=0$
*b-normalization*	p∣d and p=2	monic, P(0)=0, if $2^{i}\leq d\leq 2^{i+1}-3$ for some *i*
		then either $a_{r}=0$ or $a_{r-1}=0$, where $r=2^{i}-1$

**Table 3:** Number of PPs for degree 12 polynomials over GF(q).

*q*	$N_{12}\left(q\right)$
17	68,126,982,656
19	46,631,675,376
23	13,755,394,444

## Data Availability

The original contributions presented in this study are included in the article. Further inquiries can be directed to the corresponding author.
